# Supplementary material for: Glucosylation of Isoeugenol and Monoterpenes in Corynebacterium glutamicum by YdhE from Bacillus lichenformis
Source: Molecules. 2023 Apr 28;28(9):3789. doi: 10.3390/molecules28093789 (PMC10180135; doi:10.3390/molecules28093789)
Supplement: Supplementary file 1 [file molecules-28-03789-s001.zip › molecules-2332698-supplementary.pdf]

Glucosylation of Isoeugenol and Monoterpenes in *Corynebacterium glutamicum* by YdhE from *Bacillus lichenformis*

Su Yeong Ma [1,†], Obed Jackson Amoah [1,†], Hue Thi Nguyen [1] and Jae Kyung Sohng [1,2,*]

1 Department of Life Science and Biochemical Engineering, Sun Moon University, 70 Sun Moon-ro 221, Tangjeong-myeon, Asan-si 31460, Republic of Korea

2 Department of Pharmaceutical Engineering and Biotechnology, Sun Moon University, 70 Sun Moon-ro 221, Tangjeong-myeon, Asan-si 31460, Republic of Korea

* Correspondence: sohng@sunmoon.ac.kr; Tel.: +82-(41)-530-2246; Fax: +82-(41)-530-8229

† These authors contributed equally to this work.

Supplementary data

Figure S1. ^{1}H (700 MHz), ^{13}C (176 MHz) and COSY NMR Spectral Data of isoeugenol-1-O-β-D-glucoside (**1b**) in dimethyl sulfoxide-d_6. (**A**) ^{1}H NMR, (**B**) ^{13}C NMR, and (**C**) COSY.

Figure S2. ^{1}H (700 MHz), ^{13}C (176 MHz) and COSY NMR Spectral Data of isoeugenol-1-O-β-D-(2"-acetyl)-glucoside (**1c**) in dimethyl sulfoxide-d_6. (**A**) ^{1}H NMR, (**B**) ^{13}C NMR, and (**C**) COSY.

Figure S3. Water solubility test of isoeugenol. (**A**) isoeugenol standard, (**B**) isoeugenol (**1a**), isoeugenol-1-O-β-D-(2"-acetyl)-glucoside (**1c**), and isoeugenol-1-O-β-D-glucoside (**1b**) in ethyl acetate fraction, and (**C**) **1b** and **1c** in aqueous (water) layer fraction

Figure S4. Structures of monoterpene substrates used in this study.

Figure S5. In vivo bioconversion of eugenol (**2a**) to eugenol glucoside (**2b**) and eugenol acetylated glucoside (**2c**) in *C. glutamicum* pSKSM-YdhE.

(**A**) HPLC chromatogram analysis in *C. glutamicum* pSKSM-YdhE., (**B**) Whole-cell biotransformation with pSK003 in *C. glutamicum.* (**C**) Eugenol standard (1 mM), (**D**) UV spectra of **2a** and glucoside derivatives **2b** and **2c**.

Figure S6. In vivo bioconversion of thymol (**3a**) to thymol glucoside (**3b**) in *C. glutamicum* pSKSM-YdhE.

(A) HPLC chromatogram analysis in *C. glutamicum* pSKSM-YdhE., (**B**) Whole-cell biotransformation with pSK003 in *C. glutamicum.* (**C**) Thymol standard (1 mM). (**D**) UV spectra of **3a** and glucoside derivative **3b**

Figure S7. In vivo bioconversion of carvacrol (**4a**) to carvacrol glucoside (**4b**) in *C. glutamicum* pSKSM-YdhE.

(A) HPLC chromatogram analysis in *C. glutamicum* pSKSM-YdhE., (**B**) Whole-cell

biotransformation with pSK003 in *C. glutamicum*. (**C**) Carvacrol standard (1mM). (**D**) UV spectra of **4a** and glucoside derivative **4b**

Figure S8. HR−QTOF ESI/MS analysis of (**A**) eugenol glucoside (**2b**); (**B**) UV/VIS of **2b**; (**C**) eugenol acetylated glucoside (**2c**); (**D**) UV/VIS of **2c**; (**E**) thymol glucoside (**3b**); (**F**) UV/VIS of **3b**; (**G**) carvacrol glucoside (**4b**); (**H**) UV/VIS of **4b**.

Figure S1

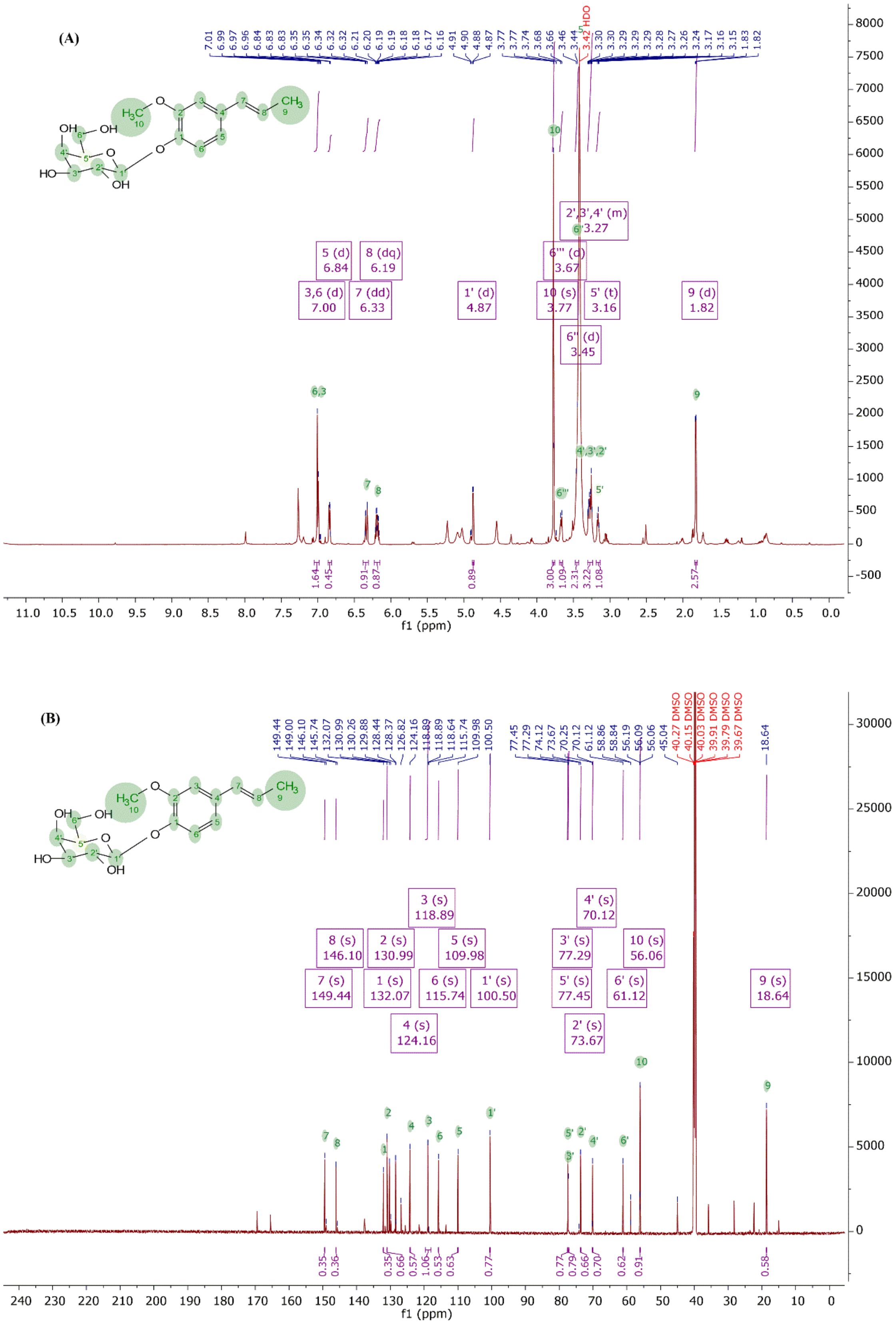
(A)
7.01 6.99 6.97 6.96 6.84 6.83 6.83 6.35 6.35 6.34 6.32 6.32 6.21 6.20 6.19 6.19 6.19 6.18 6.18 6.17 6.16 4.91 4.90 4.88 4.87 3.77 3.77 3.74 3.68 3.66 3.46 3.44 3.42 HDO 3.30 3.30 3.29 3.29 3.29 3.28 3.27 3.26 3.24 3.17 3.16 3.15 1.83 1.82
5 (d) 6.84
8 (dq) 6.19
3,6 (d) 7.00
7 (dd) 6.33
1' (d) 4.87
2',3',4' (m) 3.27
6''' (d) 3.67
10 (s) 3.77
5' (t) 3.16
6'' (d) 3.45
9 (d) 1.82
1.64 0.45 0.91 0.87 0.89 3.00 1.09 2.31 3.22 1.08 2.57
f1 (ppm)
(B)
149.44 149.00 146.10 145.74 132.07 130.99 130.26 129.88 128.44 128.37 126.82 124.16 118.89 118.89 118.64 115.74 109.98 100.50 77.45 77.29 74.12 73.67 70.25 70.12 61.12 58.86 58.84 56.19 56.09 56.06 45.04 40.27 DMSO 40.15 DMSO 40.03 DMSO 39.91 DMSO 39.79 DMSO 39.67 DMSO 18.64
3 (s) 118.89
4' (s) 70.12
8 (s) 146.10
2 (s) 130.99
5 (s) 109.98
3' (s) 77.29
10 (s) 56.06
7 (s) 149.44
1 (s) 132.07
6 (s) 115.74
1' (s) 100.50
5' (s) 77.45
6' (s) 61.12
9 (s) 18.64
4 (s) 124.16
2' (s) 73.67
0.35 0.36 0.35 0.66 0.57 1.06 0.53 0.63 0.77 0.77 0.79 0.66 0.70 0.62 0.91 0.58
f1 (ppm)

(C)

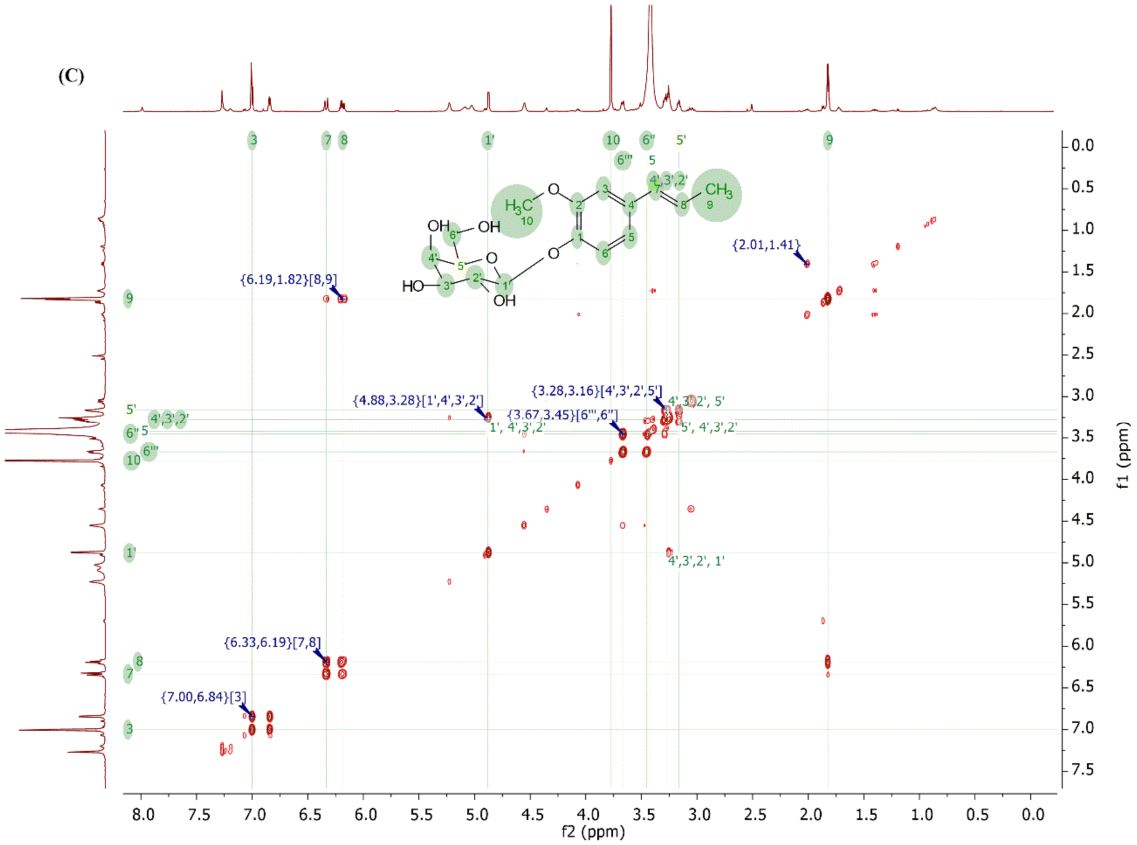

{6.19,1.82}[8,9]
{2.01,1.41}
{4.88,3.28}[1',4',3',2']
{3.28,3.16}[4',3',2',5']
{3.67,3.45}[6''',6'']
{6.33,6.19}[7,8]
{7.00,6.84}[3]
4',3',2', 5'
1', 4',3',2'
5', 4',3',2'
4',3',2', 1'
f2 (ppm)
f1 (ppm)

Figure S2

(A)

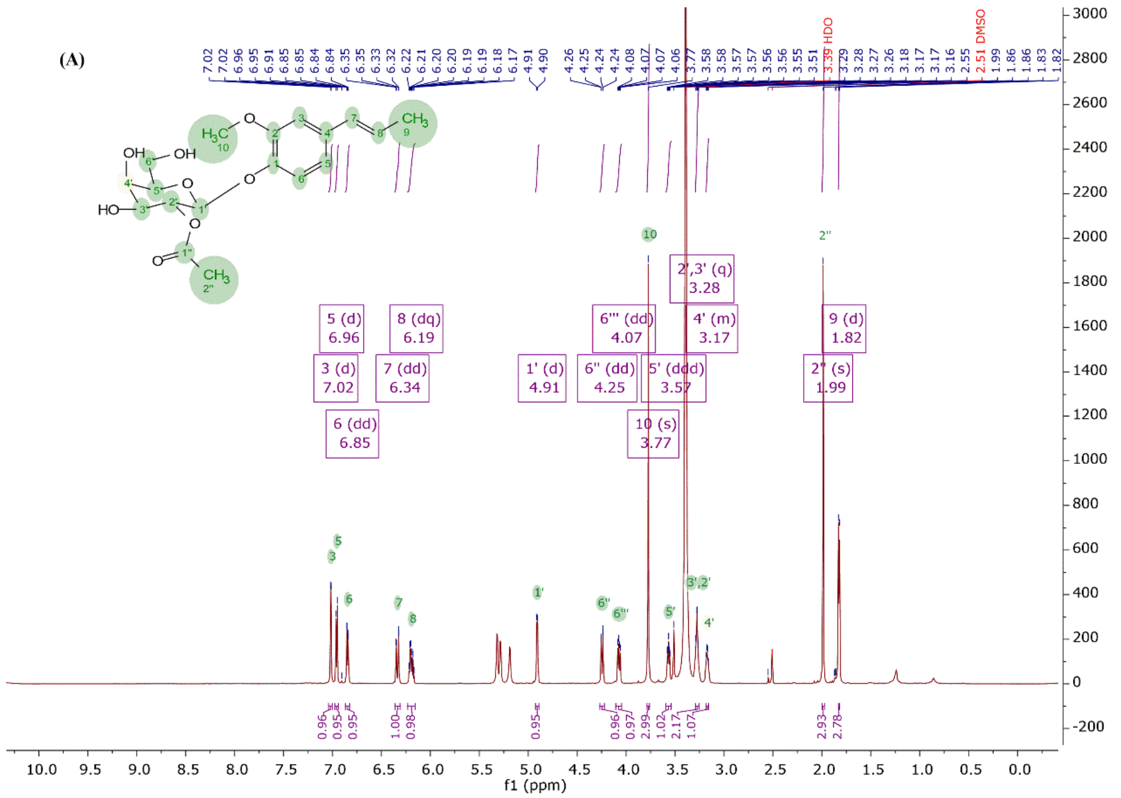
3 (d)
7.02
5 (d)
6.96
6 (dd)
6.85
7 (dd)
6.34
8 (dq)
6.19
1' (d)
4.91
6'' (dd)
4.25
6''' (dd)
4.07
10 (s)
3.77
5' (ddd)
3.57
2',3' (q)
3.28
4' (m)
3.17
2'' (s)
1.99
9 (d)
1.82
HDO
DMSO
f1 (ppm)

(B)

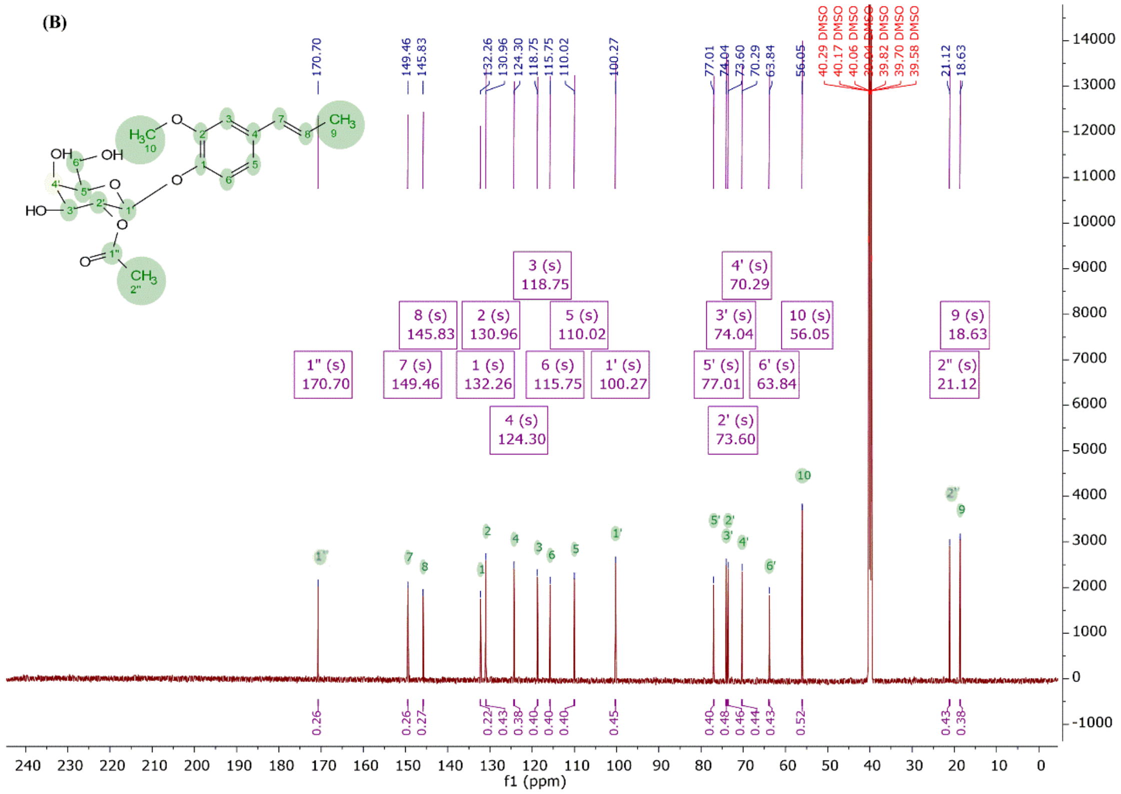
1'' (s)
170.70
7 (s)
149.46
8 (s)
145.83
1 (s)
132.26
2 (s)
130.96
4 (s)
124.30
3 (s)
118.75
6 (s)
115.75
5 (s)
110.02
1' (s)
100.27
5' (s)
77.01
3' (s)
74.04
2' (s)
73.60
4' (s)
70.29
6' (s)
63.84
10 (s)
56.05
2'' (s)
21.12
9 (s)
18.63
DMSO
f1 (ppm)

(C)

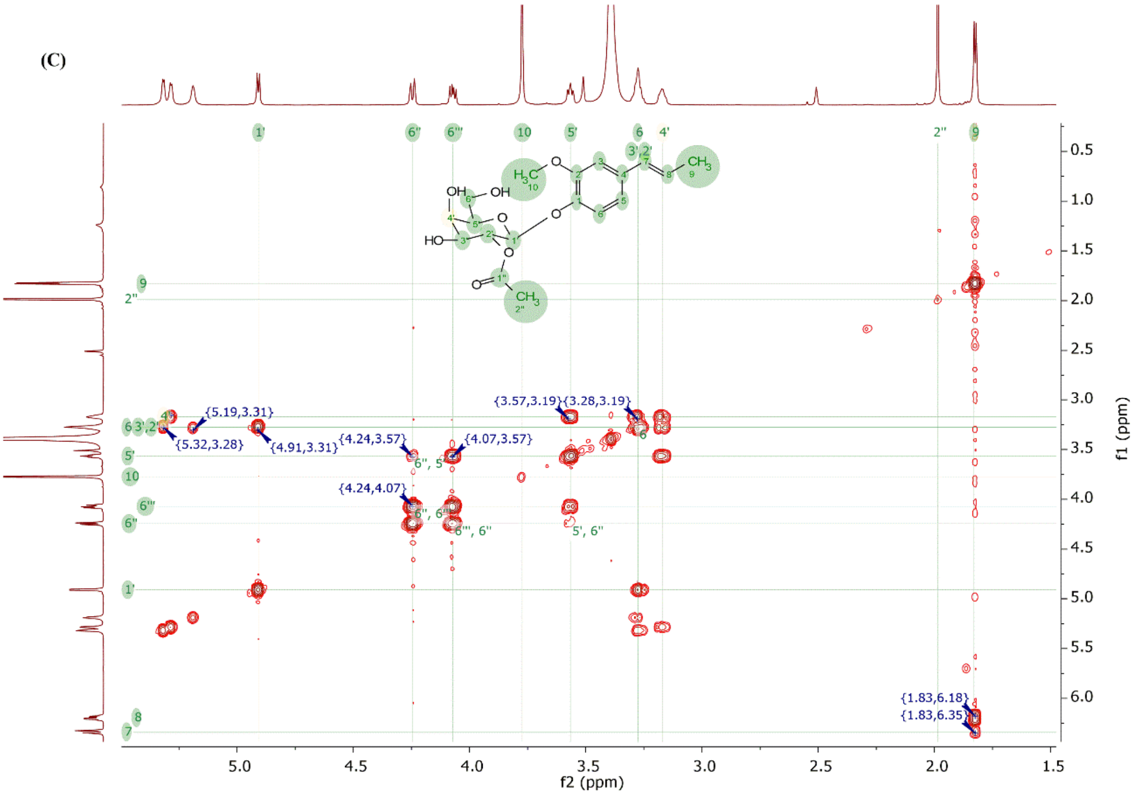

{5.19,3.31}
{5.32,3.28}
{4.91,3.31}
{4.24,3.57}
{4.07,3.57}
{4.24,4.07}
{3.57,3.19}
{3.28,3.19}
{1.83,6.18}
{1.83,6.35}
f2 (ppm)
f1 (ppm)

Figure S3

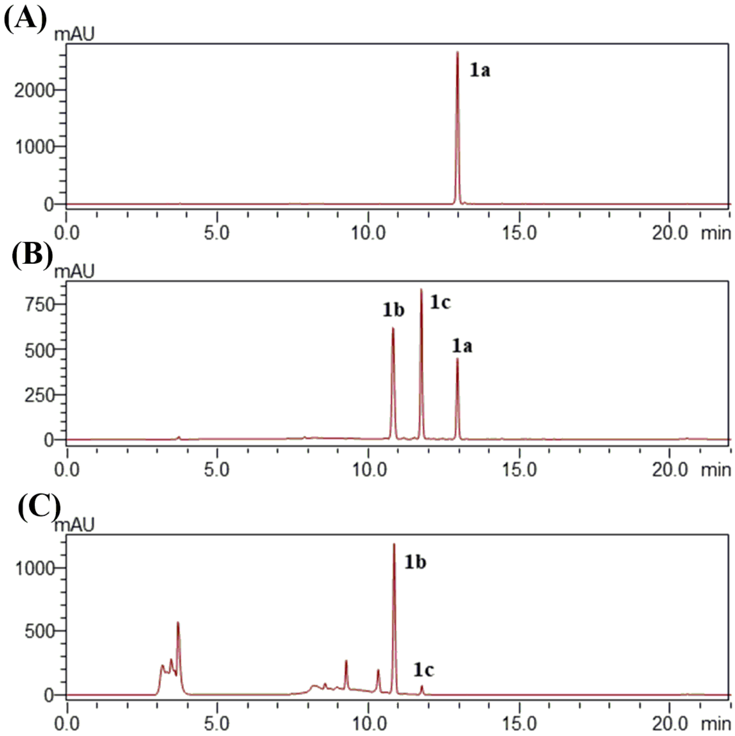
(A)
mAU
2000
1000
0
1a
0.0
5.0
10.0
15.0
20.0
min
(B)
mAU
750
500
250
0
1b
1c
1a
0.0
5.0
10.0
15.0
20.0
min
(C)
mAU
1000
500
0
1b
1c
0.0
5.0
10.0
15.0
20.0
min

Figure S4

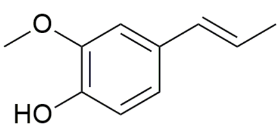

Isoeugenol (1a)

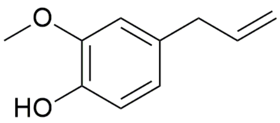

Eugenol (2a)

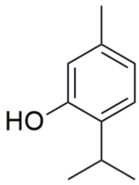

Thymol (3a)

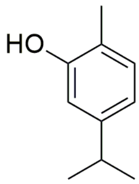

Carvacrol (4a)

Figure S5

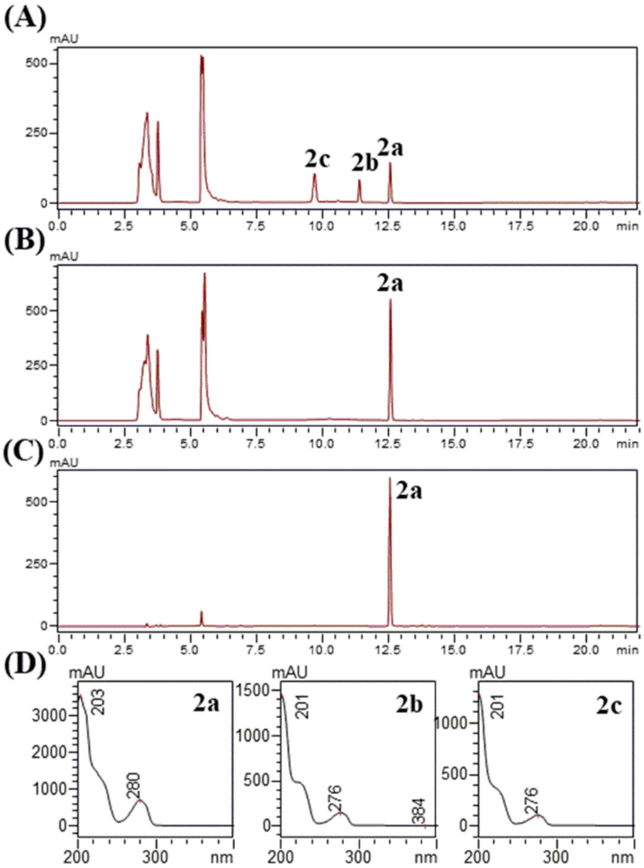
(A)
mAU
500
250
0
2c
2b
2a
0.0
2.5
5.0
7.5
10.0
12.5
15.0
17.5
20.0
min
(B)
mAU
500
250
0
2a
(C)
mAU
500
250
0
2a
(D)
mAU
3000
2000
1000
0
203
280
2a
200
300
nm
1500
1000
500
201
276
384
2b
201
276
2c

Figure S6

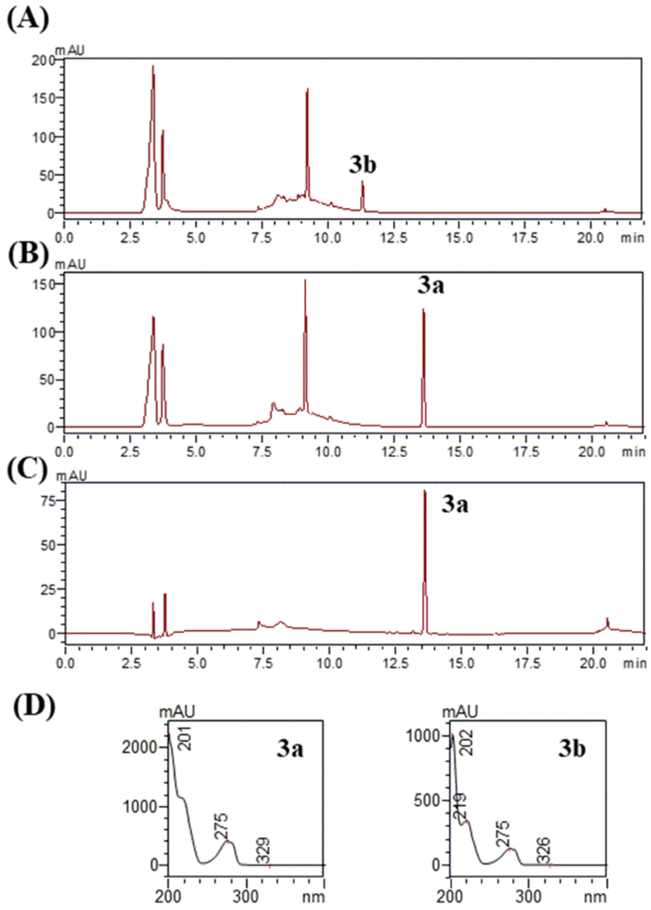
(A)
mAU
200
150
100
50
0
3b
0.0
2.5
5.0
7.5
10.0
12.5
15.0
17.5
20.0
min
(B)
mAU
150
100
50
0
3a
0.0
2.5
5.0
7.5
10.0
12.5
15.0
17.5
20.0
min
(C)
mAU
75
50
25
0
3a
0.0
2.5
5.0
7.5
10.0
12.5
15.0
17.5
20.0
min
(D)
mAU
2000
1000
0
201
275
329
3a
200
300
nm
mAU
1000
500
0
202
219
275
326
3b
200
300
nm

Figure S7

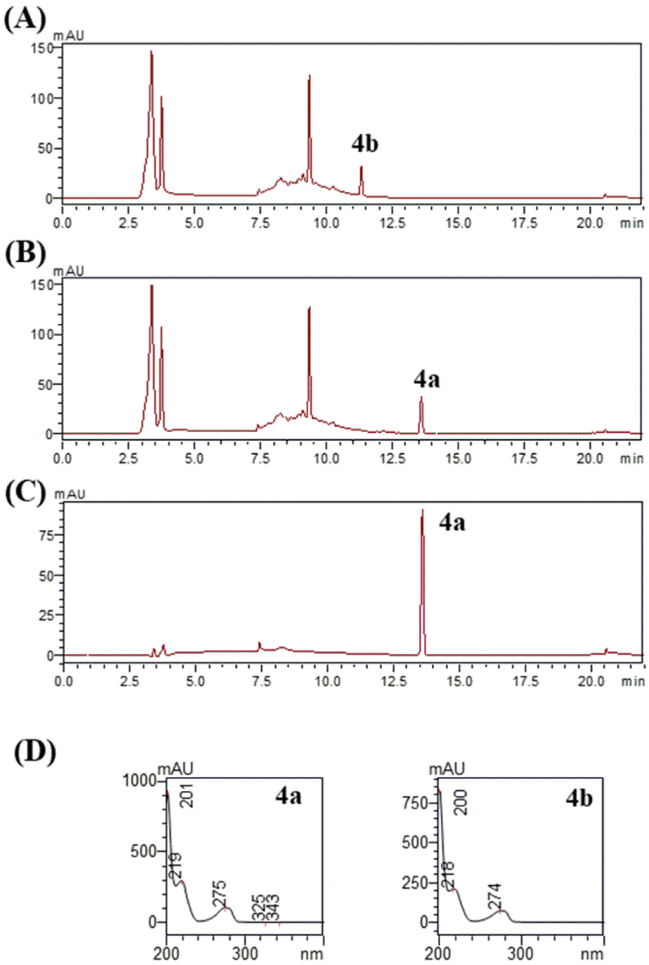
(A)
mAU
4b
min
(B)
mAU
4a
min
(C)
mAU
4a
min
(D)
mAU
4a
201
219
275
325
343
nm
mAU
4b
200
218
274
nm

Figure S8

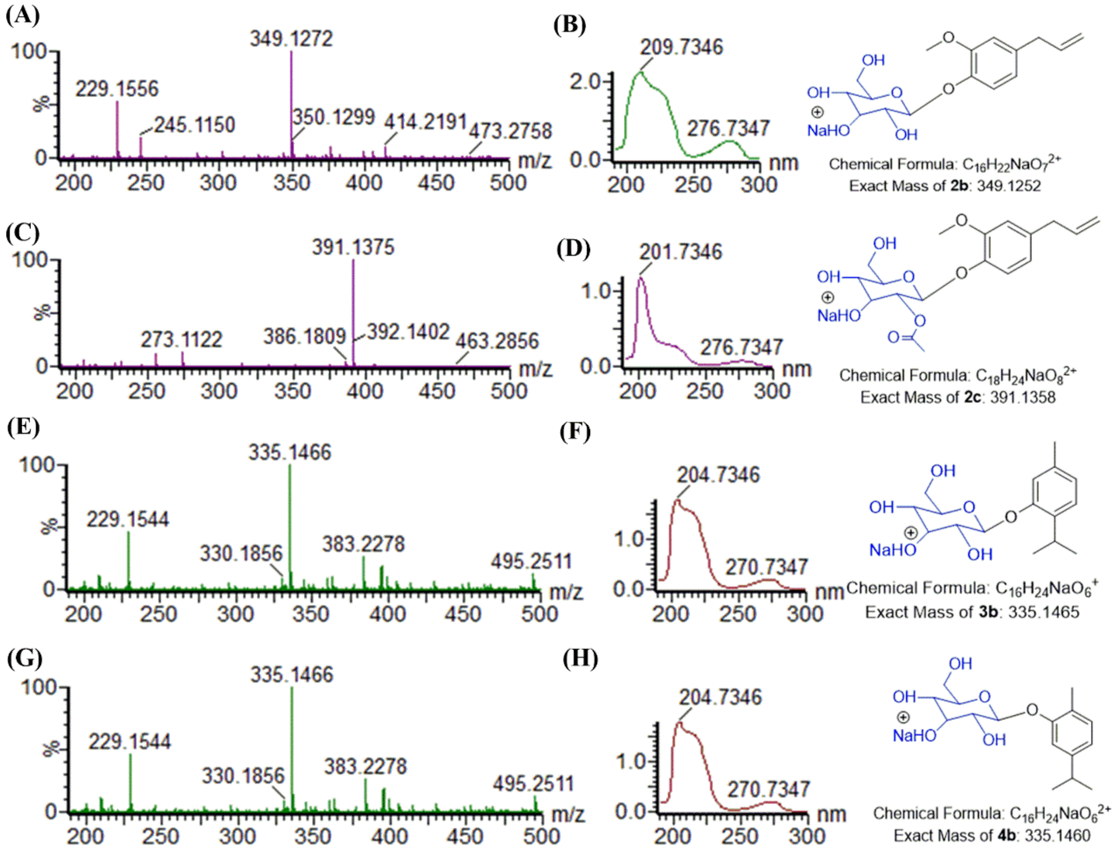

(A)
349.1272
229.1556
245.1150
350.1299
414.2191
473.2758
m/z
(B)
209.7346
276.7347
nm
Chemical Formula: C16H22NaO7 2+
Exact Mass of 2b: 349.1252
(C)
391.1375
273.1122
386.1809
392.1402
463.2856
m/z
(D)
201.7346
276.7347
nm
Chemical Formula: C18H24NaO8 2+
Exact Mass of 2c: 391.1358
(E)
335.1466
229.1544
330.1856
383.2278
495.2511
m/z
(F)
204.7346
270.7347
nm
Chemical Formula: C16H24NaO6 +
Exact Mass of 3b: 335.1465
(G)
335.1466
229.1544
330.1856
383.2278
495.2511
m/z
(H)
204.7346
270.7347
nm
Chemical Formula: C16H24NaO6 2+
Exact Mass of 4b: 335.1460
